# *Post mortem* MRI of cholinergic white matter pathways across neurodegenerative diseases

**DOI:** 10.1002/alz.71851

**Published:** 2026-09-21

**Authors:** Francisco J. López‐González, Milan Nemy, Cene Jerele, Andrés Jiménez‐Pérez, María José López‐Martínez, Laura Jonkman, Alberto Rábano, Pascual Sánchez‐Juan, Michel J. Grothe, Daniel Ferreira

**Affiliations:** ^1^ Reina Sofia Alzheimer Center, CIEN Foundation, ISCIII Madrid Spain; ^2^ Division of Clinical Geriatrics, Center for Alzheimer Research, Department of Neurobiology, Care Sciences and Society Karolinska Institutet Stockholm Sweden; ^3^ Department of Cognitive Systems and Neurosciences, Czech Institute of Informatics, Robotics and Cybernetics Czech Technical University in Prague Prague Czech Republic; ^4^ Faculty of Medicine University of Ljubljana Ljubljana Slovenia; ^5^ Department of Anatomy and Neurosciences Amsterdam UMC Amsterdam the Netherlands; ^6^ Program Neurodegeneration and Brain Imaging Amsterdam Neuroscience Amsterdam the Netherlands; ^7^ Facultad de Ciencias de la Salud Universidad Fernando Pessoa Canarias Las Palmas Spain

**Keywords:** Alzheimer's disease, Cholinergic Pathways Hyperintensities Scale, cholinergic white matter pathways, Lewy body disease, *post mortem* magnetic resonance imaging

## Abstract

**INTRODUCTION:**

Cholinergic white matter pathway (CWMP) degeneration is central in Alzheimer's disease (AD) and Lewy body disease (LBD). CWMP degeneration can be assessed in vivo using magnetic resonance imaging (MRI) proxies, but neuropathological validation is limited.

**METHODS:**

We studied *post mortem* in situ 3T MRIs of 55 brain donors with standardized neuropathologic assessment (AD, LBD, AD+LBD, other dementias, controls). CWMP integrity was assessed quantitatively using diffusion tensor imaging and visually using the Cholinergic Pathways Hyperintensities Scale (CHIPS) on fluid‐attenuated inversion recovery MRI.

**RESULTS:**

CWMP diffusivity was strongly associated with CHIPS (*p < 0.001*), independent of global white matter damage. AD and AD+LBD showed greater CWMP degeneration than LBD, other dementias, and controls (*p *< 0.05). Multivariate analyses across neuropathological variables identified hippocampal sclerosis and vascular co‐pathology as strongest predictors for CWMP degeneration.

**DISCUSSION:**

CWMP degeneration is pronounced in AD and mixed AD+LBD pathology and is additionally affected by vascular co‐pathology and hippocampal sclerosis. CHIPS may serve as a clinically accessible MRI‐based proxy of CWMP integrity.

## Highlights

- *Post mortem* magnetic resonance imaging (MRI) reveals cholinergic white matter (WM) degeneration across dementias.
- Cholinergic WM degeneration is greater in Alzheimer's disease and mixed pathology.
- Hippocampal sclerosis and vascular pathology contribute to cholinergic damage.
- Cholinergic MRI measures show specificity beyond global WM lesion burden.
- A visual cholinergic WM scale aligns with diffusion‑derived degeneration.

## 1 BACKGROUND

The cholinergic system has been extensively investigated in healthy aging and neurodegenerative diseases such as Alzheimer's disease (AD) and Lewy body disease (LBD).^1^, ^2^, ^3^ One of the key structures within the cholinergic system is the nucleus basalis of Meynert (NBM). Cholinergic neuronal loss in the NBM is associated with tau and amyloid beta (Aβ) accumulation, highlighting a critical role of the NBM in disease progression.^4^ On magnetic resonance imaging (MRI), volume loss of the NBM has been reported in patients with AD and LBD,^5^ even at pre‐dementia stages of these diseases.^6^, ^7^

The NBM provides widespread cholinergic innervation to the cerebral cortex via the cingulum and external capsule white matter (WM) pathways. Traditional diffusion tensor imaging (DTI) studies have demonstrated a correlation between reduced NBM volume and decreased WM microstructural integrity across widespread WM areas.^8^ More recently, targeted diffusion MRI tractography approaches have enabled the reconstruction and quantification of NBM‐associated cholinergic WM pathways in vivo. Using these advanced tractography‐based techniques, several studies have demonstrated a selective degeneration of cholinergic WM pathways in AD and LBD.^9^, ^10^ This degeneration has been replicated in subsequent studies, demonstrating disruption of cholinergic WM pathways across dementia and pre‐dementia stages of AD and LBD in several independent cohorts.^11^, ^12^ However, all this evidence is derived from imaging studies without autopsy confirmation, leaving uncertainty about the pathological substrates underlying the observed diffusion changes. The only *post mortem* DTI study to date provided critical validation by confirming that diffusion‐derived metrics within MRI‐based cholinergic WM pathways correlate with local neuronal loss in corresponding basal forebrain regions in AD.^13^ Yet, the neuropathological correlates of cholinergic WM degeneration across distinct neurodegenerative diseases remain unexplored.

Another challenge is that both advanced diffusion MRI tractography and *post mortem* assessments remain largely research tools and are not applicable in routine clinical practice. Recently, a study demonstrated a strong correlation between in vivo MRI‐based measures of cholinergic WM pathways and a clinically available visual rating scale for cholinergic WM degeneration—the Cholinergic Pathway Hyperintensities Scale (CHIPS).^14^ This finding suggests the possibility of assessing cholinergic pathway–related WM degeneration in clinical settings using standard MRI. Unlike the widely used Fazekas scale, which provides a global assessment of non‐specific WM lesion burden,^15^ the CHIPS focuses on WM regions traversed by cholinergic projections and may therefore serve as a pathway‐informed proxy of cholinergic WM degeneration, although it does not directly measure cholinergic integrity.

Despite these important seminal studies, the neuropathological correlates of MRI‐based cholinergic WM pathways and CHIPS remain unexplored across neurodegenerative diseases. With a focus on AD and LBD, the current study aimed to assess multimodal *post mortem* in situ MRI to quantify cholinergic WM pathways and evaluate CHIPS in neuropathologically confirmed donors with AD, LBD, mixed AD+LBD pathology, other neurodegenerative diseases, and cognitively unimpaired controls. The first aim was to test whether diffusion‐derived measures of cholinergic WM pathways are more strongly associated with WM damage in cholinergic areas (CHIPS) than in global WM (Fazekas scale). For this aim we took advantage of a unique *post mortem* MRI set up to examine the association between mean diffusivity (MD) in cholinergic pathways and CHIPS, which has not previously been investigated in neuropathologically confirmed donors. The second aim was to compare cholinergic WM pathways across diagnostic groups (AD, LBD, mixed AD+LBD, other neurodegenerative diseases, and control donors). The third aim was to determine which specific neuropathological features, irrespective of diagnostic group, best predict cholinergic WM pathway integrity. The included neuropathological features were AD pathology, LBD pathology, vascular pathology, hippocampal sclerosis (HS), transactive response DNA binding protein 43 kDa (TDP‐43), argyrophilic grain disease, and aging‐related tau astrogliopathy.

This study combines multimodal *post mortem* MRI with detailed neuropathological data to demonstrate how different pathologies contribute to degeneration of cholinergic WM pathways across major neurodegenerative conditions, bridging the gap between imaging biomarkers and their neuropathological underpinnings in an end‐stage, advanced‐age dementia cohort characterized by extensive pathologic burden.

**RESEARCH IN CONTEXT**

1. **Systematic review**: Previous in vivo magnetic resonance imaging (MRI) studies established markers of cholinergic white matter pathway integrity and demonstrated their relevance in Alzheimer's disease (AD) and dementia with Lewy bodies. A single *post mortem* study existed only for AD, leaving the cross‐disease pathological specificity of cholinergic pathway degeneration unresolved.
2. **Interpretation**: Using multimodal *post mortem* MRI and neuropathologic measurements across autopsy‐confirmed cases with AD, Lewy body disease, mixed pathology, other neurodegenerative conditions, and controls, this study shows that cholinergic white matter degeneration is most pronounced in AD and mixed pathology. MRI markers were specifically associated with cholinergic white matter damage rather than with global white matter damage. Hippocampal sclerosis and vascular pathology were important predictors of cholinergic pathway degeneration.
3. **Future directions**: Larger datasets and longitudinal in vivo studies are needed to evaluate clinical utility, refine mechanistic models, and determine how co‐pathologies shape cholinergic pathway degeneration across neurodegenerative diseases.

## 2 METHODS

### 2.1 Subjects

We used *post mortem* in situ brain MRI of donors from the brain bank Banco de Tejidos Fundación CIEN (BT‐CIEN) in Madrid, Spain. We identified all MRI scans collected between 2012 and 2023, resulting in 85 cases. Of these, 30 were excluded from the current study due to: (1) extreme brain atrophy precluding the accurate performance of automated MRI quantification methods (*n* = 18), (2) low quality of diffusion MRI data (*n* = 9), or (3) neuropathologic diagnoses not fitting the diagnostic groups selected for this study (see below; that is, donors with Huntington (*n* = 1) or vascular (*n* = 2) diagnoses). Exclusions due to severe brain atrophy or low diffusion MRI quality were determined through expert visual quality control, blinded to any clinical or pathological information and prior to any statistical analysis. Cases were excluded when visual inspection indicated that extreme atrophy or major diffusion artifacts rendered MRI preprocessing or quantitative analysis unreliable.

The final sample included 55 brain donors, of whom 19 were recruited through the Vallecas Alzheimer's Reina Sofía (VARS) cohort.^16^, ^17^ VARS is a longitudinal observational cohort study based at a nursing home for people with dementia receiving clinical and neuroimaging assessments during life as well as *post mortem* MRI and neuropathologic assessment after death. The remaining 36 cases included in the study were external brain donors who underwent *post mortem* MRI before autopsy at the BT‐CIEN. This cohort represents individuals at advanced stages of neurodegenerative disease, with severe end‐stage pathological involvement. Consent for the use of brain tissue and medical records for research purposes was obtained, and the study was approved by the local ethics committee and conducted in accordance with the Declaration of Helsinki.

### 2.2 Neuropathological procedures and assessment

Neuropathological procedures for brain extraction and immediate processing at BT‐CIEN have been described previously.^18^, ^19^ Briefly, shortly after extraction, the brain is weighed, the left hemisphere is fixed in 4% buffered formaldehyde and cut in coronal slices for tissue block dissection after 3 to 4 weeks of fixation. The assessed brain areas are standardized in the BT‐CIEN and were not chosen specifically for the current study. Please see Table S1 in supporting information for a list of the assessed brain areas. From the tissue blocks, 4 µm sections were obtained for hematoxylin and eosin and immunohistochemical staining using primary antibodies against Aβ (GeneTex GTX27501 beta amyloid [1–40] antibody [BAM‐10]), phospho‐tau AT100 (phospho‐tau [Thr212, Ser214] monoclonal antibody [AT100, Invitrogen]), ubiquitin (ab7254 Mouse monoclonal [Ubi‐1] to Ubiquitin, Abcam), total α‐synuclein (Novocastra monoclonal antibody NCL‐L‐ASYN, clone KM51, Leica Biosystems), and total TDP‐43 (10782‐2‐AP‐150UL TDP‐43 polyclonal antibody, ProteinTech). The mean *post mortem* interval between death and brain extraction was 5.4 ± 3.7 hours.

The probability of AD neuropathological change (ADNC) was determined following guidelines from the National Institute on Aging,^20^ which integrate semiquantitative scores for amyloid plaque distribution, neurofibrillary tangle stage, and neuritic plaque density. Each component is graded (A: Thal phase, B: Braak stage, C: Consortium to Establish a Registry for Alzheimer's Disease score), and these are combined into an ADNC level from 0 to 3, where 0 indicates no AD pathology and 3 represents a high likelihood of ADNC. LBD was assessed using Braak α‐synuclein (Basyn) staging, ranging from 0 to 6.^21^ Cerebrovascular pathology was staged following Deramecourt et al.,^22^ with scores ranging from 0 to 20. For the purpose of the current study, a score of ≥ 8 was classified as positive for cerebrovascular pathology, consistent with our group's previous work using the same staging approach.^23^ HS was assessed following our recently proposed staging system (0–IV) including early stages,^18^ evaluated in both hippocampal head and body sections. Donors presenting stages > 0 in any of these two sections were classified as positive for HS. TDP‐43 proteinopathy was evaluated both with the staging system proposed by Josephs et al.^24^ and through the limbic‐predominant age‐related TDP‐43 encephalopathy (LATE) staging by Nelson et al.^25^ Donors with amygdalar inclusions (Josephs and LATE stages > 0) were categorized as positive for TDP‐43. Argyrophilic grain disease was assessed for its presence or absence, for which cases with detected argyrophilic grains were classified as positive for this disease.^26^ Aging‐related tau astrogliopathy^27^ was also assessed for its presence or absence, and presence coded as positive for this tau pathology.

### 2.3 Study groups

Most of the previous studies on cholinergic WM pathways investigated AD and LBD populations.^9^, ^10^, ^11^, ^12^, ^13^, ^14^ Hence, our main focus was on AD and LBD and, to address the second aim of the study, that is, to compare changes across diagnostic groups, participants were classified into groups according to their ADNC and Basyn staging (see Table 1).

**TABLE 1:** Diagnostic groups and neuropathological definitions.

Diagnostic group	Neuropathological definition
AD (*n *= 21)	ADNC = 3 and Basyn < 4 (Basyn = 0 [*n *= 14], Basyn = 3 [*n *= 7])
LBD (*n *= 8)	ADNC < 3 and Basyn ≥ 4 (Btau = 1 [*n *= 2], Btau = III [*n *= 3], Btau = IV [*n *= 3])
Mixed AD+LBD (*n *= 14)	ADNC = 3 and Basyn ≥ 4
OD with cognitive impairment (*n *= 7)	ADNC < 3 and Basyn < 3, including: 1 amyotrophic lateral sclerosis (ALS) 1 progressive supranuclear palsy (PSP) 2 frontotemporal lobar degeneration‐TDP 1 LATE 1 cortico‐basal degeneration (CBD) 1 argyrophilic grain disease (AGD)
CTRL (*n *= 5)	ADNC < 3 and Basyn < 3; 3 ALS, 2 controls

Abbreviations: AD, Alzheimer's disease; ADNC, Alzheimer's disease neuropathological change; Basyn, Braak α‐synuclein stage; Btau, Braak tau stage; CTRL, cognitively unimpaired control; LATE, limbic‐predominant age‐related TDP‐43 encephalopathy; LBD, Lewy body disease; OD, other neurodegenerative diseases; TDP, transactive response DNA binding protein 43 kDa.

Subjects with ADNC = 3 (indicating high neuropathologic burden) and Basyn < 4 were classified in the AD group (*n *= 21); subjects with ADNC < 3 and Basyn ≥ 4 were classified in the LBD group (*n* = 8); and subjects with ADNC = 3 and Basyn ≥ 4 were included in the mixed AD+LBD group (*n* = 14). This operational classification was designed to distinguish groups based on neuropathological burdens likely to have a meaningful impact on cholinergic degeneration and allows some degree of co‐pathology in the AD and LBD groups, whereas the mixed AD+LBD group was defined to capture cases with high burden of both pathologies (see the supplementary material in supporting information for a sensitivity analysis assessing the potential impact of this group classification on the results).

Additionally, we expand the work in previous studies by investigating other neurodegenerative diseases. Specifically, the remaining donors with ADNC and Basyn values < 3 (*n* = 12) were assigned to other neurodegenerative diseases (OD, *n* = 7) based on their neuropathological findings and the presence of cognitive impairment, or to the control group (CTRL, *n* = 5) if they did not have any cognitive impairment. Cognitive impairment and clinical diagnoses were established at routine clinical visits for the 19 donors recruited through the observational cohort or collected from medical records for the 36 external brain donors when available. Table 1 shows the clinical diagnoses of donors included in the OD and CTRL groups for completeness of information. Four donors had been clinically diagnosed with amyotrophic lateral sclerosis (ALS). One of these donors had cognitive impairment and was assigned to the OD group, while the remaining three did not have any documented cognitive impairment and were thus assigned to the CTRL group.

### 2.4 Individual pathologies

For aim three in this study, which sought to identify the neuropathological features that best predict cholinergic WM pathway integrity, we modeled individual pathologies and their combination across donors in the entire sample (*n* = 55), irrespective of diagnostic group. Specifically, we analyzed AD pathology, LBD pathology, vascular pathology, HS, TDP‐43, argyrophilic grain disease, and aging‐related tau astrogliopathy. All these variables were analyzed in their dichotomous form (positive vs. negative) as explained in section 2.2. ADNC was dichotomized and considered positive when ADNC = 3. Basyn was dichotomized and considered positive when there was α‐synuclein in at least one of the sections in olfactory bulb, amygdala, brainstem, limbic, or neocortex. Statistical details on the modeling are described in section 2.9.

### 2.5 Acquisition of *post mortem* MRI data

*Post mortem* brain MRI data were acquired using in situ (brain still in cranium) scanning on a whole‐body 3T MR scanner (Signa HDxt General Electric) with a phased‐array 8‐channel head coil. T1‐weighted images were obtained using a 3D sagittal fast spoiled gradient recalled (FSPGR) sequence with the following parameters: repetition time (TR)/echo time (TE)/inversion time (TI) = 10/4/600 ms, flip angle = 12°, and a voxel resolution of 1 × 0.469 × 0.469 mm^3^. Axial 2D fluid‐attenuated inversion recovery (FLAIR) images were acquired with TR/TE/TI = 9000/130/2100 ms, and a voxel resolution of 0.937 × 0.937 × 3.4 mm^3^. DTI was acquired with an axial 2D echo‐planar imaging sequence with diffusion gradients applied in 15 (*n* = 28), 45 (*n* = 26), or 60 (*n *= 1) non‐collinear directions, TR/TE = 8200 to 12000/95 ms, b = 1000 s/mm^2^, and a voxel resolution of 2.6 × 2.6 × 2.6 mm^3^. DTI datasets acquired with different diffusion gradient schemes (i.e., 15, 45, or 60 directions) were combined for analyses, as previous studies demonstrated that scalar diffusion metrics, such as the mean diffusivity (MD) used in our study, are minimally influenced by the number of directions.^28^ Moreover, diffusion gradient schemes were evenly distributed across groups, and are therefore unlikely to have influenced the results from a specific study group (Fisher exact test, *p* = 0.477 for 15 gradients vs. 45 gradients across groups; excluding the single case with 60 gradients for the Fisher exact test).

### 2.6 CHIPS and Fazekas scales for cholinergic and global WM degeneration

An experienced radiology resident (C.J.) scored FLAIR images using the CHIPS^29^ and Fazekas^15^ scales for cholinergic and global WM lesion burden, respectively, as previously described.^14^ In a recent study, this radiologist has demonstrated an almost perfect inter‐rater agreement with a second radiologist (weighted kappa* = *0.93).^14^ In our current study, we investigated the association of CHIPS with a fully automated method for cholinergic WM damage (MD in cholinergic pathways from tractography analysis), thus testing the reliability of CHIPS with an external method. Therefore, we chose not to additionally compare CHIPS scores to those from a second radiologist for inter‐rater reliability.

All CHIPS and Fazekas scores were performed blinded to any information about the study participants. FLAIR images were not available for two donors, and CHIPS and Fazekas were scored on T2 images comparable to the FLAIR sequence described in section 2.5.

The CHIPS scale assesses cholinergic WM lesions in the cingulum and the external capsule on four different standardized axial levels: lower external capsule, upper external capsule, corona radiata, and semioval center. Assessments were made by separating each level into anterior and posterior regions. Each of the eight regions was scored on a scale of 0 to 2 (0 = normal, 1 = < 50% extent of lesion; 2 = ≥ 50% extent of lesion). Additionally, to account for the decrease in cholinergic fiber density as the level ascends, a multiplicative factor was applied as in previous studies:^4^ 4 for the lowest level (lower external capsule) and 1 for the highest (semioval center). Each hemisphere was assessed independently, with a maximum possible score of 50.

Although we had a focus on CHIPS in this study, we also included the Fazekas scale to have a measurement of global WM lesion burden. In other words, we wanted to assess specific cholinergic WM lesions (CHIPS) in the context of global WM damage (Fazekas). Fazekas provides an assessment of lesions in widespread periventricular and deep WM regions. Periventricular Fazekas scores ranged from 0 to 3 (0 = absence; 1 = caps or pencil‐thin lining; 2 = smooth halo; 3 = irregular periventricular WM hyperintensities extending into the deep WM). Deep Fazekas scores also ranged from 0 to 3 (0 = absence; 1 = punctuated foci; 2 = beginning confluence of foci; 3 = large confluent areas). In cases of score discrepancies between these regions, the highest periventricular or deep score was used.

### 2.7 Structural MRI volumetric analysis

High‐resolution T1‐weighted structural *post mortem* MRI images were processed using the Computational Anatomy Toolbox (CAT12), implemented in SPM12, using a fully automated volumetric pipeline.^30^ Images underwent bias‐field correction, tissue segmentation, and non‐linear spatial normalization to Montreal Neurological Institute (MNI) space, with modulation applied to preserve regional volumetric information. Total intracranial volume (TIV) was estimated for each participant and used for normalization purposes.

Volumes of the cholinergic basal forebrain (cBF) were extracted using previously published stereotactic masks developed by Grothe et al.^31^, ^32^

Quality control of the automated segmentation outputs was performed using CAT12 procedures. In two donors, segmentation was unsuccessful; these cases were therefore excluded from analyses involving cBF measures, while they were retained for all other analyses.

### 2.8 Tractography analysis of cholinergic WM pathways

**FIGURE 1:**
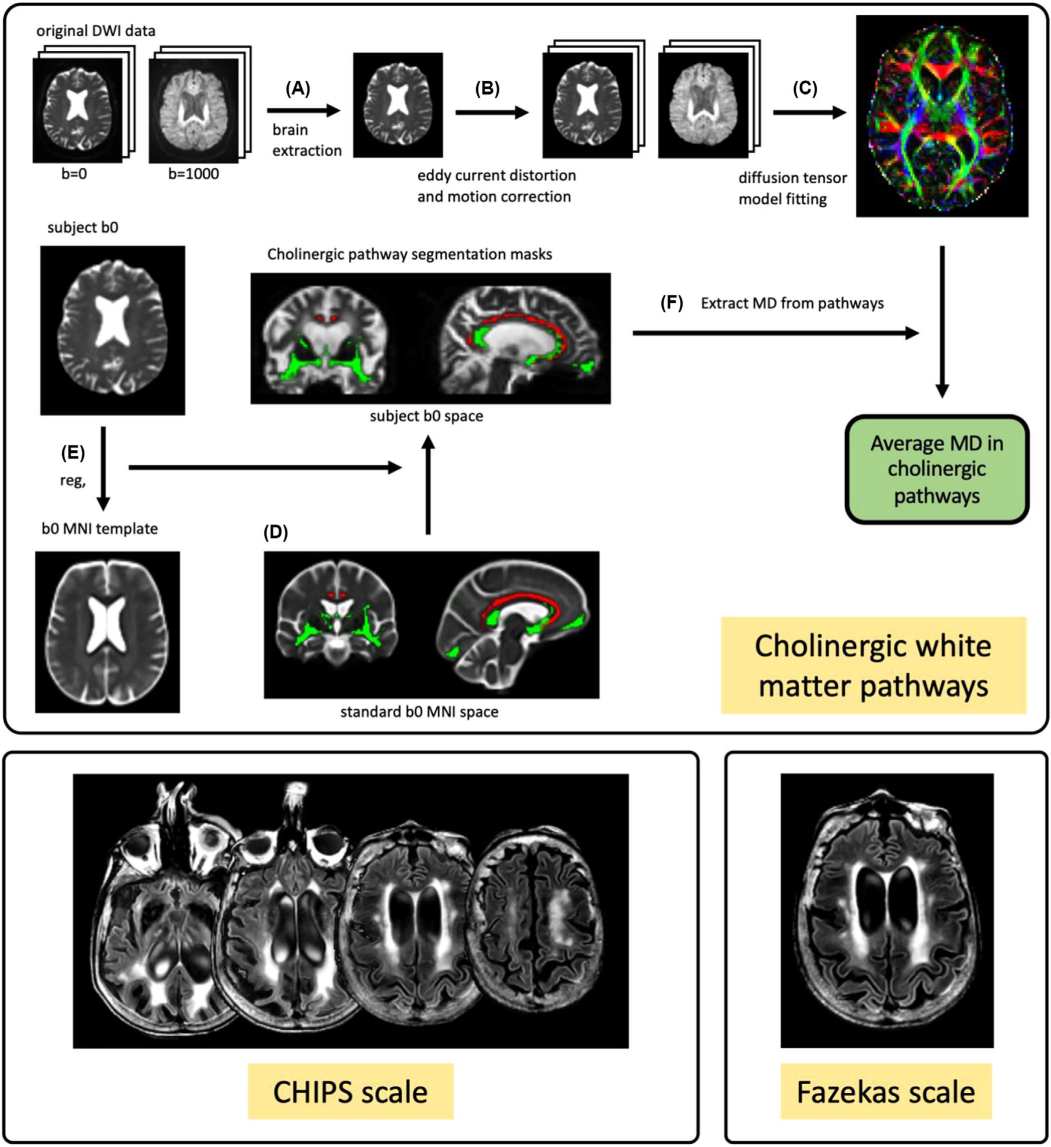
Overview of imaging analysis and clinical rating scales in an example brain donor from the mixed AD+LBD group (88‐year‐old woman). Top panel, diffusion MRI processing and quantification of cholinergic white matter pathways, with steps A–F corresponding to the processing pipeline described in the main text. The final output is the average MD in cholinergic pathways. Bottom left panel, CHIPS scoring illustrated on axial FLAIR sequence MRI images (total CHIPS score = 68). Bottom right panel, global white matter hyperintensity burden rated as Fazekas grade 3. AD, Alzheimer's disease; b0, non‐diffusion‐weighted (b = 0 s/mm^2^) image; CHIPS, Cholinergic Pathway Hyperintensities Scale; DWI, diffusion‐weighted imaging; FLAIR, fluid‐attenuated inversion recovery; LBD, Lewy body disease; MD, mean diffusivity; MNI, Montreal Neurological Institute; MRI, magnetic resonance imaging.

Diffusion MRI data were pre‐processed following the procedure described in a previous study.^33^ Briefly, non‐brain tissue was removed from each individual's b0 image (Figure 1A),^34^ eddy currents and head motion were corrected (Figure 1B),^35^ and MD maps were estimated for each donor using the FMRIB Diffusion Toolbox in FSL (Figure 1C).^36^ Next, we used established segmentation masks of cholinergic WM pathways (i.e., cingulum and external capsule pathways, Figure 1D) generated through tractography analyses with an cytoarchitectonic map of the NBM as the seed region.^33^ These segmentation masks or cholinergic pathways were transformed from standard MNI space to each donor's b0 image in native space (Figure 1E) using the non‐linear SyN registration algorithm.^37^ Microstructural properties of the cholinergic WM pathways were then estimated by averaging MD values within the back‐transformed masks in native space (Figure 1F). A threshold of 0.002 mm^2^/s was applied to exclude voxels potentially affected by partial volume effects from cerebrospinal fluid (CSF) in regions adjacent to the lateral ventricles. This threshold was determined empirically for the current cohort to minimize CSF contamination.

### 2.9 Statistical analysis

Prior to statistical analyses, all variables were assessed for normal distribution using the Shapiro–Wilk test and visual inspection of quantile‐quantile (Q‐Q) plots.

For aim one in this study, that is, to assess the relationship between cholinergic pathway MD and region‐specific versus global WM damage, the associations among MD values, cBF volumes, and CHIPS were assessed using the Pearson correlation coefficient (*r*). The proportion of variance explained (*R^2^*) was calculated as a measure of effect size. Associations among MD values, cBF volumes, and Fazekas scores (three levels) were assessed using the Kruskal–Wallis test, with epsilon‐squared (*ε^2^*) calculated as a non‐parametric measure of effect size. Both *R^2^* and *ε^2^* are expressed as the proportion of variance explained, enabling direct comparisons of the strength of association for CHIPS versus Fazekas. We also used multiple linear regression to assess the capacity of CHIPS to independently predict MD values or cBF volumes when Fazekas was simultaneously entered in the model. This allowed us to test whether MD within cholinergic WM pathways or cBF volumes are more closely associated with CHIPS than with global WM lesion burden (Fazekas) in this *post mortem* cohort. All analyses for aim one were performed on all diagnostic groups combined into a single study population.

For aim two, that is, to assess group differences in cholinergic WM pathways, we conducted group‐wise comparisons across all diagnostic groups (AD, LBD, mixed AD+LBD, OD, and CTRL) using the Kruskal–Wallis test for demographic variables, Fazekas scores, and CHIPS scores. Then, pairwise post hoc comparisons were performed using the Mann–Whitney *U* test. For binary variables (e.g., sex and presence of pathology), we report counts and percentages per diagnostic group and refrained from performing statistical testing due to small cell sizes. Between‐group differences in MD and cBF were assessed with two‐sided Mann–Whitney *U* tests. For each pairwise comparison we report the rank‐biserial correlation (*r_rb_*) with 95% confidence intervals and the corresponding *p* value. Results for MD are shown separately for the cingulum and external capsule. Where age‐adjusted analyses are presented, MD and cBF residuals were obtained by regressing MD and cBF on age prior to testing.

For aim three, that is, to explore the neuropathological determinants of cholinergic WM integrity, random forest (RF) regression models were used to assess the individual and joint contributions of different neuropathologies to cholinergic WM pathway degeneration. The outcome variable was MD in the cingulum and external capsule pathways, modeled separately. Predictor variables included AD pathology (ADNC = 3), LBD pathology (presence of α‐synuclein in at least one of the sections in olfactory bulb, amygdala, brainstem, limbic, or neocortex), vascular pathology, HS, TDP‐43, argyrophilic grain disease, and aging‐related tau astrogliopathy, along with sex and age at exitus. For this analysis, all neuropathological variables were coded as binary (positive vs. negative). The dataset was randomly divided into a training set comprising 75% of the analytical sample and a held‐out test set comprising the remaining 25%. RF hyperparameters were selected within the training set using grid search with repeated 5‐fold cross‐validation with three repetitions, using the root mean square error (RMSE) as the optimization metric. The evaluated hyperparameters included a maximum number of features of 5, 7, or 9, and a maximum tree depth of 3, 10, 20, or unrestricted. The model with the optimal hyperparameters was subsequently refitted using the complete training set. Model performance was evaluated exclusively on the held‐out test set using RMSE and *R* ^2^, which, respectively, quantify the average prediction error and the proportion of variance in MD values explained by the model. Permutation importance was estimated on the training set by quantifying the increase in RMSE after randomly permuting each predictor. Permutation was repeated five times for each predictor, and the mean importance and standard deviation were reported. Because permutation importance was estimated on the training set and some predictors were correlated, these importance estimates were interpreted as exploratory measures of model reliance.

Additionally, we report pairwise correlation coefficients among all pathology variables included in the RF analysis. These correlation analyses were performed on original variables instead of the binarized variables. Therefore, the Pearson correlation was used for pairs of continuous variables, while the Spearman correlation was applied in all other cases that included binary variables (positive vs. negative). For sensitivity analyses, we repeated these correlation analyses including age at exitus as a covariate (partial correlations).

All statistical analyses were conducted using Python (version 3.12). Data visualization was performed using the seaborn library,^38^ while statistical testing was carried out using pingouin,^39^ statsmodels,^40^ and scipy.stats.^41^ RF analyses were performed using the scikit‐learn library,^42^ which implements the RF algorithm as originally proposed by Breiman.^43^ No correction for multiple comparisons was applied to post hoc pairwise analyses. Results were deemed statistically significant at two‐tailed *p* < 0.05.

## 3 RESULTS

### 3.1 Cohort characteristics

Table 2 summarizes the key demographic characteristics, MRI‐based measures, and neuropathologic data across diagnostic groups. Age at exitus differed significantly between groups. The AD group was older than both the LBD and OD groups, whereas the CTRL group was younger than all other diagnostic groups. Sex distribution also varied across groups, with the LBD group showing the highest proportion of men and the AD group the lowest, while the remaining groups presented intermediate proportions. CHIPS and Fazekas scores were higher in the AD and mixed AD+LBD groups compared to the LBD and CTRL groups. No other significant group differences were observed for these measures. Neuropathology data for vascular pathology, HS, TDP‐43, argyrophilic grain disease, and aging‐related tau astrogliopathy are reported in Table 2. Some co‐pathology can be observed in all diagnostic groups.

**TABLE 2:** Demographic, imaging, and neuropathological characteristics for the diagnostic groups included in this study.

	AD	LBD	Mixed AD+LBD	OD	CTRL	Group differences
**n**	21	8	14	7	5	
**Age (years)**	87.9 ± 8.7, [82.1–92.8]	79.5 ± 6.2, [76.9–85.0]	82.4 ± 9.5, [80.6–88.8]	78.8 ± 8.7, [71.6–85.9]	60.3 ± 9.3, [57.1–61.6]	*H(4) *= 19.7, *p *< 0.001, AD > LBD (*p *= 0.011), AD > OD (*p *= 0.023), CTRL < rest (*p *< 0.02)
**Sex, male (%)**	5 (23.8)	5 (71.4)	6 (42.9)	4 (57.1)	3 (60.0)	
**CHIPS score**	64.5 ± 22.6	39.1 ± 20.4	64.3 ± 16.9	52.9 ± 33.1	27.6 ± 14.4	H(4) = 15.7, *p *= 0.003, AD > LBD (*p *= 0.022), AD > CTRL (*p *= 0.003), LBD < Mixed AD+LBD (*p* = 0.009), CTRL < Mixed AD+LBD (*p *= 0.003)
**Fazekas score**	3 [3–3]	2 [1.75–2.25]	3 [3–3]	2 [1.5–3]	1 [1–2]	*H(4) *= 19.0, *p *< 0.001, AD > LBD (*p *= 0.012), AD > CTRL (*p *< 0.001), LBD < Mixed AD+LBD (*p* = 0.011), Mixed AD+LBD > CTRL (*p *= 0.001)
**Vascular pathology, *n* (%)**	9 (42.9)	0 (0.0)	7 (50.0)	1 (14.3)	0 (0.0)	
**HS, *n* (%)**	17 (81.0)	0 (0.0)	8 (57.1)	3 (42.9)	0 (0.0)	
**TDP‐43, *n* (%)**	16 (76.2)	2 (25.0)	6 (50.0)	4 (57.1)	3 (60.0)	
**AGD, *n* (%)**	1 (4.8)	0 (0.0)	0 (0.0)	1 (14.3)	0 (0.0)	
**ARTAG, *n* (%)**	13 (61.9)	2 (25.0)	7 (50.0)	2 (28.6)	0 (0.0)	

*Note*: Age is reported as mean ± SD [Q1–Q3], CHIPS scores as mean ± SD, and Fazekas scores as median [Q1–Q3]. Binary variables are presented as counts of positive cases (percentage). Group differences in age, CHIPS, and Fazekas scores were assessed using the Kruskal–Wallis test followed by post hoc Mann–Whitney *U* tests; only significant pairwise differences are reported.

Abbreviations: AD, Alzheimer's disease; AGD, argyrophilic grain disease pathology; ARTAG, aging‐related tau astrogliopathy; CHIPS, Cholinergic Pathways Hyperintensities Scale; CTRL, control donors; HS, hippocampal sclerosis; LBD, Lewy body disease; Mixed AD+LBD, mixed Alzheimer's disease and Lewy body disease; OD, other neurodegenerative diseases; SD, standard deviation; TDP‐43, transactive response DNA binding protein 43 kDa pathology.

### 3.2 The association of cholinergic WM pathways and cBF with CHIPS and Fazekas scores

**FIGURE 2:**
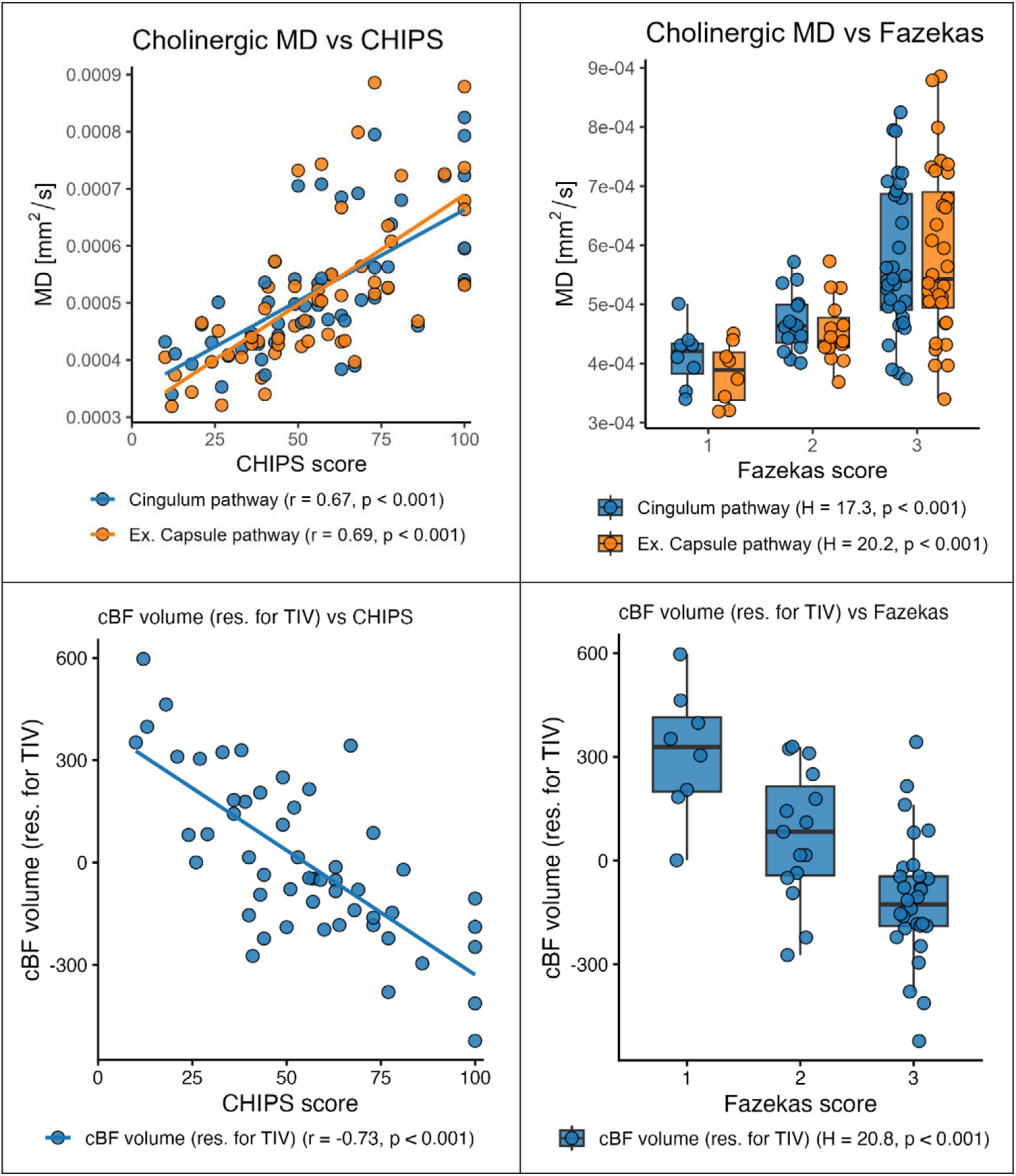
Association of cholinergic WM pathways and cholinergic basal forebrain volumes with CHIPS and Fazekas scores. Top row, Pearson correlation between MD values in cholinergic WM pathways and total CHIPS scores (left), and Kruskal–Wallis test results for differences in MD across Fazekas score categories (right), shown as boxplots with individual data points. Bottom row, Pearson correlation between cBF volume and total CHIPS scores (left), and Kruskal–Wallis test results for differences in cBF volume across Fazekas score categories (right). For the cBF analyses, cBF volume was adjusted for TIV by using residuals obtained from a linear regression of cBF volume on TIV prior to statistical testing. None of the donors showed a Fazekas score = 0 in this cohort primarily representing subjects at end stage dementia. cBF, cholinergic basal forebrain; CHIPS, Cholinergic Pathways Hyperintensities Scale; MD, mean diffusivity; TIV, total intracranial volume; WM, white matter.

For the first aim in this study, that is, comparing cholinergic‐specific versus global WM associations, we examined the association between diffusion‐derived MD in cholinergic WM pathways and CHIPS in this neuropathologically confirmed *post mortem* cohort. As another proxy of cholinergic integrity, we also examined the association of structural MRI‐based measures of cBF volume with MD values and CHIPS scores. We found a significant positive correlation between MD values in cholinergic WM pathways and total CHIPS scores. As illustrated in Figure 2, higher MD values were associated with a greater burden of cholinergic lesions as reflected by CHIPS (cingulum pathway, *r* = 0.67, *p* < 0.001; external capsule pathway, *r* = 0.69, *p* < 0.001). Similarly, cBF volume showed strong associations with CHIPS scores and MD values in cholinergic WM pathways (CHIPS: *r = *−0.73, *p* < 0.001; cingulum pathway: *r = *−0.58, *p* < 0.001; external capsule pathway: *r = *−0.60, *p* < 0.001). MD values in cholinergic WM pathways and cBF volumes also differed across Fazekas levels (cingulum pathway: *H*[2] = 17.3, *p* < 0.001; external capsule pathway: *H*[2] = 20.2, *p* < 0.001; cBF volume: *H*[2]* = *20.8, *p* < 0.001). However, effect sizes demonstrated that CHIPS explained a greater proportion of variance than Fazekas scores in both MD (cingulum: *R^2^* = 0.44 vs. *ε^2^* = 0.29; external capsule: *R^2^* = 0.48 vs. *ε^2^* = 0.35) and cBF measures (*R^2^ = *0.54 vs. *ε^2^ = *0.38). In the multiple linear regression models including both CHIPS and Fazekas scores as predictors of MD in cholinergic WM pathways or cBF volume, CHIPS remained a significant independent predictor of both MD (cingulum: β = 2.92×10^−^^6^, *p* < 0.001; external capsule: β = 3.30×10^−^^6^, *p* < 0.001) and cBF volumes (β = −5.81, *p* < 0.001), whereas Fazekas scores were no longer significantly associated with either MD or cBF measures (*p* > 0.05).

### 3.3 MD in cholinergic WM pathways across diagnostic groups

**FIGURE 3:**
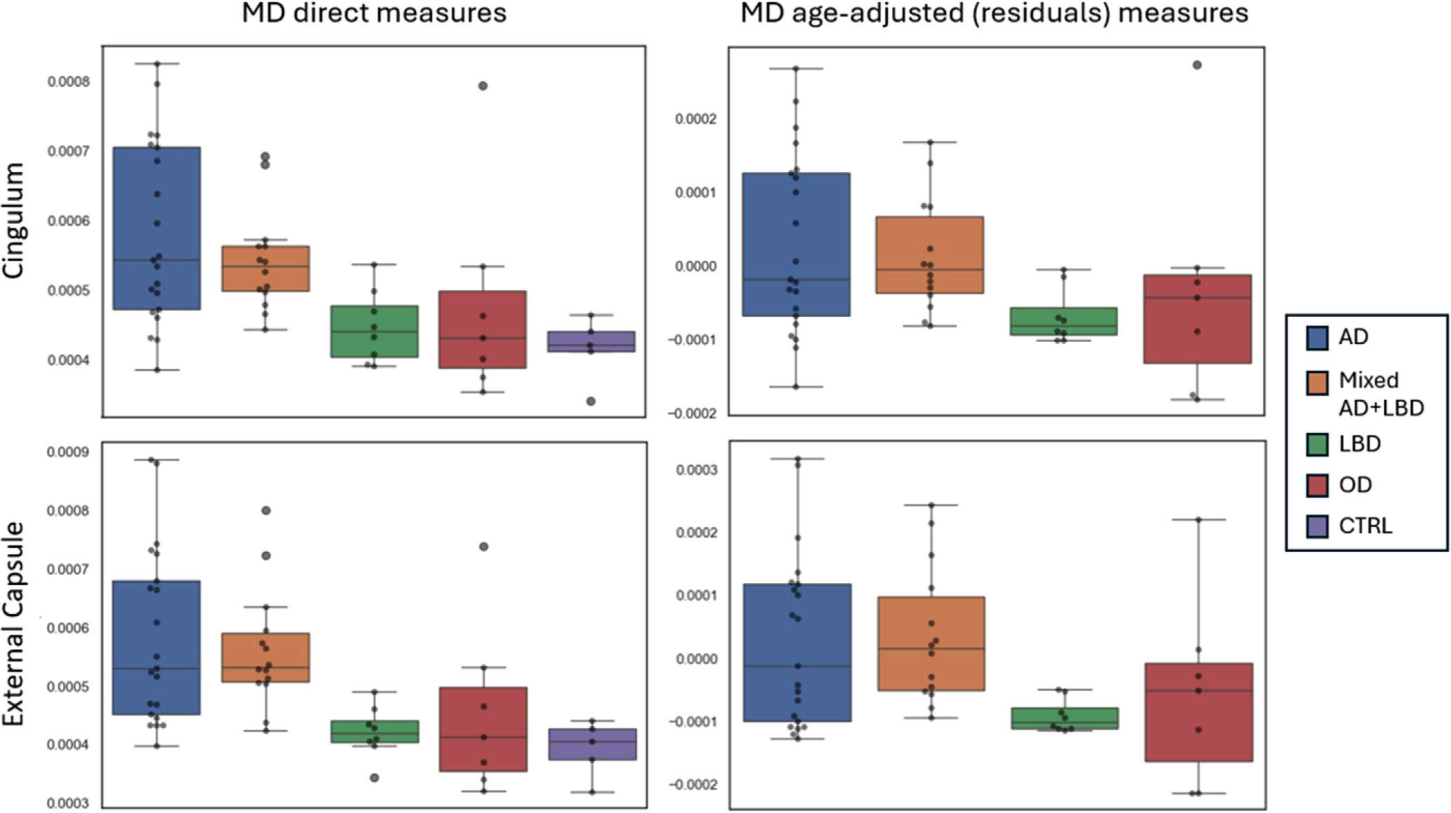
Group differences in MD values in the cingulum and external capsule pathways: direct and age‐adjusted measures (residuals). Groups: Alzheimer's disease (AD), mixed Alzheimer's disease and Lewy body disease (Mixed AD+LBD), Lewy body disease (LBD), other neurodegenerative diseases with cognitive impairment (OD) and control donors (CTRL). MD, mean diffusivity.

For the second aim in this study, the comparison across diagnostic groups, Table 3 shows significant results comparing MD in cholinergic WM pathways across diagnostic groups (the results of all comparisons are included in Table S2 in supporting information). Because there were marked group differences in age, Table 3 and Table S2 report pairwise MD comparisons both before (direct measures) and after adjusting for age (residuals), using rank‐biserial correlations to reflect effect sizes. On direct measures, both the AD and mixed AD+LBD groups showed significantly higher MD values in the cingulum and external capsule pathways than the LBD, OD, and CTRL groups. After age adjustment, the overall pattern was qualitatively similar, but effect sizes were attenuated and several contrasts no longer reached statistical significance in this small sample. Figure 3 shows MD values before and after adjusting for age. Notably, the CTRL group was excluded from age‐adjusted comparisons due to its substantially younger age (≈ 15 years), which could not be fully accounted for by correction.

**TABLE 3:** Summary table for significant differences in MD values and cBF volumes between diagnostic groups.

Group comparisons	Rank‐biserial correlation [95% CI] to reflect effect sizes, *p* value					
	MD value (Cingulum pathway)		MD value (Ex. cap. pathway)		cBF volume	
	Direct measures	Residuals	Direct measures	Residuals	Direct measures	Residuals
**AD vs. LBD**	0.63 [0.29, 0.92], ***p *< 0.01**	0.48 [0.08, 0.83], *p *= 0.053	0.74 [0.43, 0.96], ***p *< 0.01**	0.49 [0.11, 0.82], ***p *< 0.05**	0.58 [0.14, 0.91], ***p *< 0.05**	0.45 [0.05, 0.84], *p *= 0.075
**AD vs. OD**	0.52 [0.00, 0.92], ***p *< 0.05**	0.28 [0.01, 0.78], *p *= 0.296	0.51 [0.03, 0.93], ***p *< 0.05**	0.31 [0.02, 0.78], *p *= 0.249	0.13 [0.01, 0.71], *p *= 0.651	0.01 [0.01, 0.64], *p *= 1.00
**AD vs. CTRL**	0.83 [0.56, 1.00], ***p *< 0.01**	—	0.89 [0.66, 1.00], ***p *< 0.01**	—	0.54 [0.07, 1.00], *p *= 0.075	—
**Mixed AD+LBD vs. LBD**	0.75 [0.39, 0.98], ***p *< 0.01**	0.70 [0.30, 0.96], ***p *< 0.01**	0.89 [0.66, 1.00], ***p *< 0.001**	0.84 [0.55, 1.00], ***p *< 0.001**	0.79 [0.41, 1.00], ***p *< 0.005**	0.77 [0.39, 1.00], ***p *< 0.005**
**Mixed AD+LBD vs. OD**	0.55 [0.04, 1.00], ***p *< 0.05**	0.41 [0.02, 0.90], *p *= 0.149	0.55 [0.04, 1.00], ***p *< 0.05**	0.39 [0.02, 0.88], *p *= 0.172	0.31 [0.02, 0.82], *p *= 0.287	0.33 [0.02, 0.88], *p *= 0.255
**Mixed AD+LBD vs. CTRL**	0.97 [0.83, 1.00], ***p *< 0.001**	—	0.91 [0.66, 1.00], ***p *< 0.01**	—	0.69 [0.06, 1.00], ***p *< 0.05**	—

*Notes*: Pairwise comparisons were conducted and effect sizes were expressed as the rank‐biserial correlations (r_rb_) with 95% confidence intervals, followed by the *p* values from two‐sided Mann–Whitney *U* tests. Results for MD are shown separately for the cingulum and external capsule pathways, using both direct measures and age‐adjusted residuals. For cBF volumes, both direct measures (only TIV‐adjusted) and residualized measures (additionally age adjusted) are shown. Statistically significant comparisons are indicated by P‐values printed in bold font.

Abbreviations: AD, Alzheimer's disease; cBF, cholinergic basal forebrain; CI, confidence interval; CTRL, control donors; LBD, Lewy body disease; MD, mean diffusivity; Mixed AD+LBD, mixed Alzheimer's disease and Lewy body disease; OD, other neurodegenerative diseases; TIV, total intracranial volume.

Groupwise comparisons of cBF volumes showed a comparable overall pattern (see Table 3). Specifically, the AD and mixed AD+LBD groups exhibited greater involvement than the other groups. However, effect sizes for cBF were consistently smaller than those observed for MD measures. Detailed results are provided in Table S3 in supporting information.

In a sensitivity analysis assessing the possible effect of early/incipient LBD pathology in the AD group, the overall pattern of results and direction of the main effects remained consistent with the primary analyses, indicating greater cholinergic degeneration in AD and, more prominently, mixed AD+LBD groups relative to the other diagnostic groups (see Tables S4 and S5 in supporting information).

### 3.4 Neuropathological predictors of cholinergic WM pathway integrity

**FIGURE 4:**
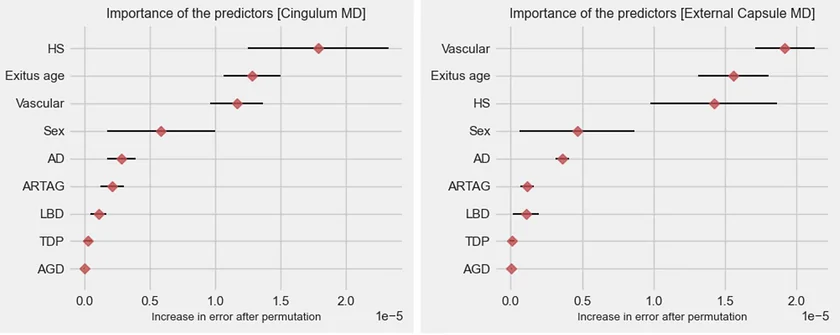
Permutation importance of predictors in the random forest regression models. MD in cingulum pathway (left) and external capsule pathway (right). Exitus age, age at exitus. AD, Alzheimer's disease; AGD, argyrophilic grain disease; ARTAG, aging‐related tau astrogliopathy; HS, hippocampal sclerosis; LBD, Lewy body disease; MD, mean diffusivity; Vascular, vascular pathology; TDP, transactive response DNA binding protein 43 kDa proteinopathy.

Regarding the third aim in this study, that is, the identification of neuropathological predictors of cholinergic WM integrity, the RF regression models revealed distinct patterns of neuropathological contributions toward the degeneration of cingulum and external capsule WM pathways (Figure 4). On the held‐out test set, the RF model for the cingulum pathway showed a good predictive performance with an explained variance of 50.2% and an RMSE of 9.17 × 10^−^^5^. Training‐set permutation importance identified HS as the most influential predictor, followed by vascular pathology. In contrast, for the external capsule pathway (held‐out test‐set performance: 41.0% of explained variance, RMSE = 1.28 × 10^−^^4^), training‐set permutation importance identified vascular pathology as the most influential predictor, followed by HS. The other assessed pathologies, including AD pathology, LBD pathology, TDP‐43, aging‐related tau astrogliopathy, and argyrophilic grain disease, had minimal contributions in both models.

To evaluate the influence of age and sex, we re‐ran both RF models excluding sex and age at exitus. We observed that although these variables contributed to the original models, their exclusion did not substantially change the overall pattern of results. Notably, the permutation importance of HS increased when age at exitus was removed in both the model predicting MD in the cingulum pathway (increase in error after permuting HS rose from 1.79 × 10^−5^ to 2.23 × 10^−5^; RMSE improved from 9.17 × 10^−^^5^ to 8.56 × 10^−^^5^; *R^2^* increased from 0.50 to 0.57) and in the model predicting MD in the external capsule pathway (increase in error after permuting HS rose from 1.42 × 10^−5^ to 1.99 × 10^−5^; RMSE improved from 1.28 × 10^−^^4^ to 1.14 × 10^−^^4^; *R^2^* increased from 0.41 to 0.54).

**FIGURE 5:**
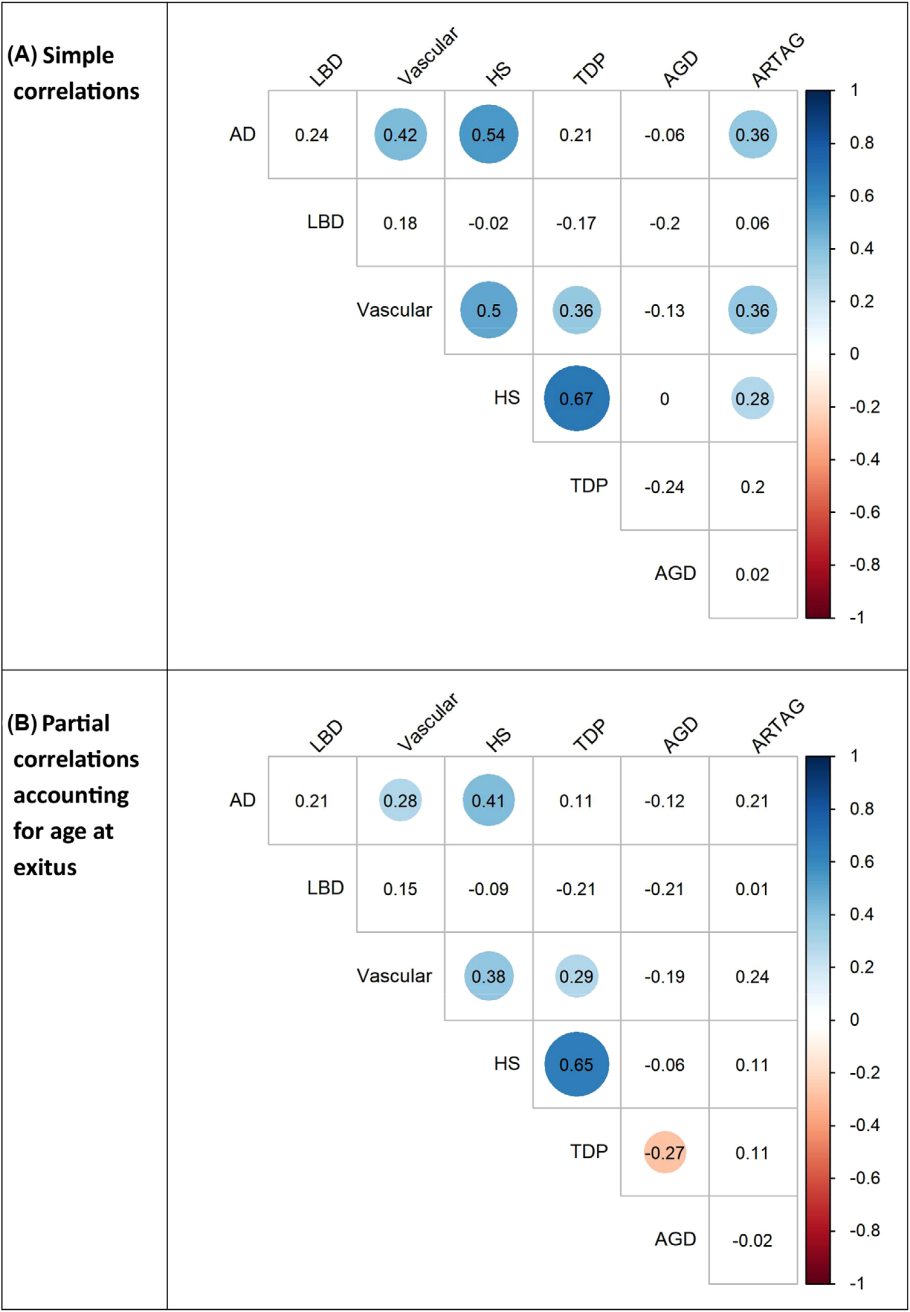
Correlation matrix for the pathology predictors of the RF models. Background of significant correlations (*p* < 0.05) was colored according to the value of the correlation coefficient (bar on the most right), otherwise left white. AD, Alzheimer's disease; AGD, argyrophilic grain disease; ARTAG, aging‐related tau astrogliopathy; HS, hippocampal sclerosis; LBD, Lewy body disease; RF, random forest; TDP, transactive response DNA binding protein 43 kDa proteinopathy; Vascular, vascular pathology.

We complemented the RF analyses with a correlation matrix for all pairs of neuropathological predictors. This was done to aid interpretation of the RF results and to further assess whether vascular pathology and HS achieved high importance scores because they were largely independent from the other pathologies (AD pathology, LBD pathology, TDP‐43, aging‐related tau astrogliopathy, or argyrophilic grain disease), or because they shared associations with them. As shown in Figure 5A, vascular pathology and HS were strongly correlated with each other, and HS also showed strong correlations with TDP‐43 and AD pathology. When adding age as a covariate in partial correlation analysis, the overall pattern of associations remained similar, but correlation coefficients became weaker (Figure 5B).

## 4 DISCUSSION

The present study extends previous in vivo diffusion MRI research that identified degeneration of cholinergic WM pathways in AD and LBD.^9^, ^10^ By combining *post mortem* MRI with detailed neuropathological data, we examined these pathways in a neuropathologically confirmed cohort and assessed their microstructural integrity in relation to established visual rating scales for cholinergic (CHIPS) and global WM (Fazekas) damage. We compared cholinergic WM pathways across pathologic groups and assessed the individual and joint contribution of different neuropathologies toward cholinergic MD degeneration. We found a stronger association of MD in cholinergic WM pathways with CHIPS than with Fazekas. Cholinergic WM degeneration was higher in the AD and mixed AD+LBD groups compared to LBD or CTRL donors. The multivariate RF model showed that hippocampal sclerosis and vascular pathology had the highest relative importance in explaining variability in cholinergic WM degeneration, exceeding the relative importance of AD, LBD, TDP‐43, aging‐related tau astrogliopathy, or argyrophilic grain disease. Additionally, we observed that effect sizes for cBF volume were consistently smaller than those observed for MD measures, suggesting a higher sensitivity of diffusion‐derived metrics to detect cholinergic system alterations at the pathway level.

The strong associations observed between *post mortem* MRI‐based cholinergic WM pathways and both CHIPS and Fazekas scores are in line with our recent in vivo study,^14^ which reported robust associations of MD in cholinergic WM pathways with both CHIPS and Fazekas scores in patients with LBD. Additionally, our current study demonstrated that CHIPS was the only significant predictor of MD in cholinergic WM pathways when Fazekas was also added to a multiple regression model. While Fazekas scores capture broader WM degeneration, the CHIPS is anatomically targeted to cholinergic WM projections, which likely accounts for this superior performance in our study. In line with this interpretation, CHIPS was also a stronger predictor of cBF volumes than Fazekas scores, representing a well‐established and pathologically validated in vivo proxy of cholinergic system degeneration. CHIPS may thus be a complementary MRI marker in studies of neurodegeneration, particularly when evaluating cholinergic degeneration. The present *post mortem* findings provide additional biological context for the association between MD alterations and CHIPS in cholinergic projection pathways. Taken together, these findings suggest that diffusion‐derived measures preferentially reflect structural vulnerability of cholinergic pathways, which appears to be more pronounced in AD and mixed pathology in our cohort.

The second aim in this study was to compare diagnostic groups. We found that the degeneration of cholinergic WM pathways was more pronounced in the AD and mixed AD+LBD groups than in the LBD group. Several previous studies have investigated degeneration of cholinergic WM pathways in AD and LBD, but they all relied on clinical diagnoses. Schumacher et al.^10^ reported a greater degeneration of cholinergic WM pathways in clinically defined AD than in clinically defined LBD. This corresponds to our current finding of greater degeneration of cholinergic WM pathways in neuropathologically defined AD and mixed AD+LBD than in LBD. In a follow‐up study using free‐water MRI,^11^ the same group reported cholinergic WM degeneration in both AD and LBD relative to healthy controls. However, the magnitude of cholinergic WM degeneration may partially depend on the assessed cohort and WM pathway methods. Lin et al.^13^ performed *post mortem* in situ MRI with neuropathological confirmation of AD but reconstructed NBM‐cortical projections rather than the specific pathways examined here. Our findings extend previous work by demonstrating that cholinergic WM pathway degeneration is most pronounced in neuropathologically confirmed AD and mixed AD+LBD compared to donors with LBD. LBD‐related cholinergic dysfunction may arise more from synaptic alterations linked to α‐synuclein pathology, which may not be accompanied by the same degree of structural WM damage.

Moreover, it is notable that the LBD group showed a low burden of vascular co‐pathology, which in our analyses emerged as a key predictor of pathway degeneration. Autopsy‐based work indicates that although cerebrovascular pathology is common in LBD, it generally exerts a weaker contribution to disease expression compared to AD. For example, in a large *post mortem* cohort, cerebrovascular disease was present in a substantial proportion of LBD cases (≈ 58%), yet its associations with clinical outcomes were less robust than in AD.^44^ These findings support the notion that, in LBD, α‐synuclein pathology and co‐occurring neurodegenerative processes represent the primary substrates, while vascular lesions are frequent but likely play a secondary role, which may help explain the comparatively lower vascular burden observed in our LBD group with advanced dementia.

Regarding the third aim in our study, the RF models in this end‐stage disease autopsy cohort showed that AD‐ and LBD‐related pathology did not rank among the strongest predictors of cholinergic WM pathway degeneration. Instead, vascular pathology and HS showed the highest relative importance in predicting cholinergic WM pathway degeneration. A previous in vivo MRI study demonstrated that vascular pathology was the main predictor of cholinergic WM pathway degeneration in cognitively unimpaired individuals, above and beyond AD biomarkers.^45^ In our current analysis, HS was the most important predictor of MD in the cingulum pathway, which could be explained by the connectivity between cingulum bundle and hippocampus.^46^ Beyond its link to limbic TDP‐43 pathology, HS is also closely related to cerebrovascular changes—particularly atherosclerosis and arteriolosclerosis—which can independently contribute to HS and may account for most cases of LATE‐NC–negative HS.^47^, ^48^ In this context, HS may index a broader microvascular substrate that could contribute to WM damage, particularly within pathways connected to the hippocampus, thereby providing a potential link between HS and WM alterations in cholinergic pathways as observed in our study. For the external capsule pathway, vascular pathology was the most important predictor of MD, consistent with its vulnerability to small vessel–related damage.^49^, ^50^ While AD pathology defines diagnostic categories and aligns with observed group differences, co‐pathologies such as HS and vascular burden may better account for individual variability in pathway integrity and degeneration. Altogether, cholinergic WM pathway degeneration may reflect cumulative and mixed pathology burden in this end‐of‐life cohort at advanced stages of disease, instead of reflecting disease‐specific processes. Taken together, our results suggest that diffusion alterations within cholinergic pathways and CHIPS scores should be interpreted as integrated markers reflecting the combined impact of cholinergic system degeneration and non‐specific vascular pathology affecting cholinergic pathways.

This study is based on end‐of‐life brain tissue, representing very advanced stages of disease. By contrast, previous in vivo studies^9^, ^10^, ^11^ have typically focused on individuals with mild to moderate dementia. In earlier stages, group‐level differences between AD and LBD may be less pronounced, whereas in advanced disease, cumulative co‐pathology burden and older age become stronger determinants of cholinergic WM degeneration. Our study clarifies those in vivo findings at later disease stages, but findings cannot be directly translated to regular research cohorts or clinical settings.

Our study has some limitations. First, detailed clinical information was not available for most brain donors. However, we designed our study to focus on *post mortem* cholinergic WM pathway quantification and pathology, and clinical correlates were not in the scope of this study. Second, the CTRL group was small, younger than the dementia groups, and included ALS donors without documented cognitive impairment. Although we statistically adjusted for age, residual confounding cannot be fully excluded and comparisons involving the CTRL group should be interpreted cautiously. While the cohort is comparable in size to other imaging‐pathologic *post mortem* studies,^13^, ^51^ some of our study groups (particularly the LBD group) may have been underpowered. Exclusions due to severe atrophy or poor MRI quality may also have introduced selection bias, thereby influencing the final composition of the sample. Moreover, post hoc pairwise comparisons were not corrected for multiple testing and should be considered exploratory pending replication in independent cohorts. Although RF model performance was evaluated on a held‐out test set, the small size of the test set may limit the precision of the performance estimates, and permutation importance was estimated on the training set. The predictor rankings should therefore be interpreted as exploratory, particularly given the correlations among neuropathological predictors. Third, diffusion MRI was acquired using a single‐shell protocol with a single non‐zero *b* value and relatively low spatial resolution, which may limit the precision of diffusion tensor estimates. *Post mortem* in situ diffusion MRI is close to the in vivo setting but some differences have been observed, most notably a reduction in diffusivity due to declining body temperature and cellular changes occurring *peri*‐ and *post mortem*. Nonetheless, *ante*‐ to *post mortem* diffusion changes appear to be linear,^52^ and it is important to consider this effect when translating *post mortem* findings to the in vivo setting. Fourth, vascular pathology was operationalized using an established global neuropathological staging score, which limits anatomical granularity and does not allow tract‐specific mapping of vascular lesions along cholinergic projection pathways. Future studies may benefit from combining high‐resolution MRI with regionally resolved neuropathological measures to examine how spatial patterns of neuropathological burden relate to cholinergic integrity, potentially using algorithmic or multivariate approaches.

In conclusion, this *post mortem* in situ MRI study demonstrates that cholinergic WM pathways are compromised in pathologically confirmed AD and AD+LBD, with greater severity than in LBD. This finding could not be fully explained by the sole impact of AD‐related pathology on cholinergic WM pathways, given that vascular pathology and HS also had an important contribution to cholinergic WM degeneration in this cohort. This emphasizes the complex interplay among multiple pathologies in people with dementia and the impact of co‐pathologies in older age and advanced disease stages. This study provides *post mortem* insight into the relationship between diffusion‐derived MD in anatomically defined cholinergic WM pathways, CHIPS and Fazekas, offering additional biological context for the clinical use of these radiological rating scales.

## CONFLICT OF INTEREST STATEMENT

D.F. consults for BioArctic and has received honoraria from Esteve Pharmaceuticals S.A. L.E.J. reports contract research with Imeka. All other authors report no competing interests. Author disclosures are available in the Supporting Information.

## CONSENT STATEMENT

Consent for the use of brain tissue and medical records for research purposes was obtained, and the study was approved by the local ethics committee and conducted in accordance with the Declaration of Helsinki.

## Supporting information



Supporting Information

Supporting Information
