# Supplementary material for: Post mortem MRI of cholinergic white matter pathways across neurodegenerative diseases
Source: Alzheimers Dement. 2026 Sep 21;22(9):e71851. doi: 10.1002/alz.71851 (PMC13593583; doi:10.1002/alz.71851)
Supplement: Supplementary file 2 [file ALZ-22-e71851-s002.pdf]

Supplementary Material:

Post-mortem MRI of cholinergic white matter pathways across neurodegenerative diseases

Supplementary Table 1. Tissue blocks for neuropathological assessment. Twenty-eight brain areas from cortical and subcortical brain regions were obtained for paraffin embedding. This set of fixed tissue samples comprises all brain regions included in current consensus criteria for the diagnosis and classification of most prevalent neuropathological conditions underlying dementia.

Olfactory bulb	Posterior hippocampus (body)
Lateral frontal cortex (CERAD criteria)	Caudate nucleus and putamen, coronal level of nucleus accumbens
Orbitofrontal cortex and underlying white matter	Lenticular nucleus and nucleus basalis of Meynert
Corpus callosum and cingulate cortex	Posterior hypothalamus, including mammillary nucleus
Precentral cortex (primary motor)	Thalamus and subthalamic nucleus
Inferior lateral parietal cortex (CERAD criteria)	Superior cerebellar cortex
Medial parietal cortex	Anterior cerebellar vermis
Lateral occipital cortex	Midbrain
Lateral temporal cortex (CERAD criteria)	Middle pons
Inferior temporal cortex (inferior temporal gyrus)	Medulla (middle portion)
Anterior insula	Cervical spinal cord
Calcarine gyrus	Periventricular region, frontal horn of lateral ventricle
Amygdala	Choroid plexus
Anterior hippocampus and entorhinal cortex	Pituitary gland

CERAD, Consortium to Establish a Registry for Alzheimer's Disease.

Supplementary Table 2. Differences in MD values between diagnostic groups. Pairwise comparisons were conducted and effect sizes were expressed as the rank-biserial correlations (r_{rb}) with 95% confidence intervals, followed by the p-values from two-sided Mann-Whitney U tests. Results are shown separately for the cingulum and external capsule pathways, using both direct measures and age-adjusted residuals.

Group comparisons	Rank-biserial correlation [95% CI] to reflect effect sizes, p-value			
	MD value (Cingulum pathway)		MD value (Ex. cap. pathway)	
	Direct measures	Residuals	Direct measures	Residuals
AD vs. LBD	0.63 [0.29, 0.92], **p<0.01**	0.48 [0.08, 0.83], p=0.053	0.74 [0.43, 0.96], **p<0.01**	0.49 [0.11, 0.82], **p<0.05**
AD vs. OD	0.52 [0.00, 0.92], **p<0.05**	0.28 [0.01, 0.78], p=0.296	0.51 [0.03, 0.93], **p<0.05**	0.31 [0.02, 0.78], p=0.249
AD vs. CTRL	0.83 [0.56, 1.00], **p<0.01**	-	0.89 [0.66, 1.00], **p<0.01**	-
Mixed AD+LBD vs. LBD	0.75 [0.39, 0.98], **p<0.01**	0.70 [0.30, 0.96], **p<0.01**	0.89 [0.66, 1.00], **p<0.001**	0.84 [0.55, 1.00], **p<0.001**
Mixed AD+LBD vs. OD	0.55 [0.04, 1.00], **p<0.05**	0.41 [0.02, 0.90], p=0.149	0.55 [0.04, 1.00], **p<0.05**	0.39 [0.02, 0.88], p=0.172
Mixed AD+LBD vs. CTRL	0.97 [0.83, 1.00], **p<0.001**	-	0.91 [0.66, 1.00], **p<0.01**	-
AD vs. mixed AD+LBD	0.11 [0.01, 0.49], p=0.59	0.01 [0.01, 0.42], p=0.987	0.02 [0.01, 0.45], p=0.933	0.11 [0.01, 0.49], p=0.602
LBD vs. OD	0.11 [0.00, 0.71], p=0.779	0.14 [0.00, 0.82], p=0.694	0.00 [0.00, 0.71], p=1.00	0.14 [0.04, 0.79], p=0.694
LBD vs. CTRL	0.25 [0.00, 0.90], p=0.524	-	0.32, [0.00, 0.90], p=0.379	-
OD vs. CTRL	0.14 [0.03, 0.83], p<0.755	-	0.20 [0.03, 0.83], p=0.639	-

MD, mean diffusivity; AD, Alzheimer's disease; LBD, Lewy body disease; AD+LBD, mixed Alzheimer's disease and Lewy body disease; OD, other neurodegenerative diseases; CTRL, control donors

Supplementary Table 3. Differences in cBF volumes between diagnostic groups. Pairwise comparisons were conducted and effect sizes were expressed as the rank-biserial correlations (r_{rb}) with 95% confidence intervals, followed by the p-values from two-sided Mann-Whitney U tests. Both direct measures (TIV-adjusted) and residualized measures (age-adjusted) are shown

Group comparisons	Rank-biserial correlation [95% CI] to reflect effect sizes, p-value	
	Direct measures	Residuals
AD vs. LBD	0.58 [0.14, 0.91], **p<0.05**	0.45 [0.05, 0.84], p=0.075
AD vs. OD	0.13 [0.01, 0.71], p=0.651	0.01 [0.01, 0.64], p=1.00
AD vs. CTRL	0.54 [0.07, 1.00], p=0.075	-
Mixed AD+LBD vs. LBD	0.79 [0.41, 1.00], **p<0.005**	0.77 [0.39, 1.00], **p<0.005**
Mixed AD+LBD vs. OD	0.31 [0.02, 0.82], p=0.287	0.33 [0.02, 0.88], p=0.255
Mixed AD+LBD vs. CTRL	0.69 [0.06, 1.00], **p<0.05**	-
Mixed AD+LBD vs. AD	0.28 [0.02, 0.65], p=0.184	0.50 [0.14, 0.80], **p<0.05**
LBD vs. OD	0.25 [0.04, 0.79], p=0.463	0.36 [0.00, 0.86], p=0.281
LBD vs. CTRL	0.25 [0.00, 0.90], p=0.524	-
OD vs. CTRL	0.54 [0.03, 1.00], p=0.149	-

cBF, cholinergic basal forebrain; MD, mean diffusivity; AD, Alzheimer's disease; LBD, Lewy body disease; AD+LBD, mixed Alzheimer's disease and Lewy body disease; OD, other neurodegenerative diseases; CTRL, control donors

Supplementary Table 4. Differences in MD values between diagnostic groups reassigning cases including the Basyn=3 cases in the mixed AD +LBD group. Pairwise comparisons were conducted and effect sizes were expressed as the rank-biserial correlations (r_{rb}) with 95% confidence intervals, followed by the p-values from two-sided Mann-Whitney U tests. Results are shown separately for the cingulum and external capsule pathways, using both direct measures and age-adjusted residuals.

Group comparisons	Rank-biserial correlation [95% CI] to reflect effect sizes, p-value			
	MD value (Cingulum pathway)		MD value (Ex. cap. pathway)	
	Direct measures	Residuals	Direct measures	Residuals
AD vs. LBD	0.55 [0.14, 0.89], **p<0.05**	0.36 [0.02, 0.79], p=0.188	0.67 [0.29, 0.96], **p<0.05**	0.32 [0.02, 0.77], p=0.238
AD vs. OD	0.46 [0.03, 0.92], p=0.10	0.18 [0.00, 0.71], p=0.535	0.43 [0.02, 0.88], p=0.128	0.14 [0.02, 0.69], p=0.636
AD vs. CTRL	0.80 [0.49, 1.00], **p<0.01**	-	0.86 [0.57, 1.00], **p<0.005**	-
Mixed AD+LBD vs. LBD	0.76 [0.50, 0.96], **p<0.001**	0.70 [0.39, 0.95], **p<0.005**	0.89 [0.71, 1.00], **p<0.001**	0.83 [0.61, 0.99], **p<0.001**
Mixed AD+LBD vs. OD	0.58 [0.07, 0.99], **p<0.05**	0.43 [0.02, 0.89], p=0.100	0.59 [0.09, 1.00], **p<0.05**	0.47 [0.05, 0.90], p=0.07
Mixed AD+LBD vs. CTRL	0.94 [0.79, 1.00], **p<0.001**	-	0.92 [0.77, 1.00], **p<0.001**	-
AD vs. mixed AD+LBD	0.19 [0.01, 0.58], p=0.36	0.30 [0.02, 0.66], p=0.143	0.28 [0.02, 0.64], p=0.178	0.4 [0.05, 0.73], **p<0.05**
LBD vs. OD	0.11 [0.00, 0.71], p=0.779	0.14 [0.00, 0.79], p=0.694	0.00 [0.00, 0.75], p=1.00	0.14 [0.04, 0.79], p=0.694
LBD vs. CTRL	0.25 [0.00, 0.90], p=0.524	-	0.32, [0.02, 0.85], p=0.379	-
OD vs. CTRL	0.14 [0.03, 0.83], p<0.755	-	0.20 [0.03, 0.77], p=0.639	-

MD, mean diffusivity; AD, Alzheimer's disease; LBD, Lewy body disease; AD+LBD, mixed Alzheimer's disease and Lewy body disease; OD, other neurodegenerative diseases; CTRL, control donors

Supplementary Table 5. Differences in cBF volumes between diagnostic groups reassigning cases including the Basyn=3 cases in the mixed AD +LBD group. Pairwise comparisons were conducted and effect sizes were expressed as the rank-biserial correlations (r_{rb}) with 95% confidence intervals, followed by the p-values from two-sided Mann-Whitney U tests. Both direct measures (TIV-adjusted) and residualized measures (age-adjusted) are shown

Group comparisons	Rank-biserial correlation [95% CI] to reflect effect sizes, p-value	
	Direct measures	Residuals
AD vs. LBD	0.54 [0.12, 0.90], **p<0.05**	0.35 [0.02, 0.81], p=0.210
AD vs. OD	0.14 [0.01, 0.76], p=0.643	0.08 [0.01, 0.65], p=0.817
AD vs. CTRL	0.57 [0.08, 1.00], p=0.075	-
Mixed AD+LBD vs. LBD	0.75 [0.32, 1.00], **p<0.005**	0.74 [0.38, 1.00], **p<0.005**
Mixed AD+LBD vs. OD	0.24 [0.01, 0.80], p=0.370	0.27 [0.01, 0.79], p=0.314
Mixed AD+LBD vs. CTRL	0.62 [0.08, 1.00], **p<0.05**	-
Mixed AD+LBD vs. AD	0.38 [0.03, 0.76], p=0.068	0.48 [0.11, 0.80], **p<0.05**
LBD vs. OD	0.25 [0.04, 0.79], p=0.463	0.36 [0.00, 0.82], p=0.281
LBD vs. CTRL	0.25 [0.00, 0.90], p=0.524	-
OD vs. CTRL	0.54 [0.03, 1.00], p=0.149	-

cBF, cholinergic basal forebrain; MD, mean diffusivity; AD, Alzheimer's disease; LBD, Lewy body disease; AD+LBD, mixed Alzheimer's disease and Lewy body disease; OD, other neurodegenerative diseases; CTRL, control donors
